# The Correlation Mechanism between Dominant Bacteria and Primary Metabolites during Fermentation of Red Sour Soup

**DOI:** 10.3390/foods11030341

**Published:** 2022-01-25

**Authors:** Xiaojie Zhou, Zhiqi Liu, Le Xie, Liangyi Li, Wenhua Zhou, Liangzhong Zhao

**Affiliations:** 1 College of Food Science and Engineering, Central South University of Forestry and Technology, Changsha 410004, China; xiaojiezhou2020@163.com (X.Z.); xiele4949@163.com (L.X.); liliangyi0088@126.com (L.L.); 2 College of Food and Chemical Engineering, Shaoyang University, Shaoyang 422000, China; LeChen0713@163.com; 3 Hunan Key Laboratory of Processed Food for Special Medical Purpose, Changsha 410004, China; 4 Hunan Provincial Key Laboratory of Soybean Products Processing and Safety Control, Shaoyang 422000, China

**Keywords:** LAB, fermentation, red sour soup, bacterial diversity, metabolites

## Abstract

Chinese red sour soup is a traditional fermented product famous in the southwestern part of China owing to its distinguished sour and spicy flavor. In the present study, the effect of inoculation of lactic acid bacteria (LAB) on the microbial communities and metabolite contents of the Chinese red sour soup was investigated. Traditional red sour soup was made with tomato and red chilli pepper and a live count (10^8^ CFU/mL) of five bacterial strains (including *Clostridium intestinalis*: *Lacticaseibacillus rhamnosus*: *Lactiplantibacillus plantarum*: *Lacticaseibacillus casei*: *Lactobacillus paracei*) was added and fermented for 30 days in an incubator at 37 °C. Three replicates were randomly taken at 0 d, 5 d, 10 d, 15 d, 20 d, 25 d and 30 d of fermentation, with a total of 21 sour soup samples. Metabolomic analysis and 16S-rDNA amplicon sequencing of soup samples were performed to determine microbial diversity and metabolite contents. Results revealed that fermentation resulted in the depletion of native bacterial strains as LAB dominated over other microbes, resulting in differences in the relative abundance of bacteria, and types or contents of metabolites. A decrease (*p* < 0.01) in Shannon and Simpson indices was observed at different fermentation times. The metabolomic analyses revealed a significant increase in the relative content of 10 metabolites (particularly lactic acid, thymine, and ascorbic acid) in fermented samples as compared to the control. The correlation network revealed a positive association of *Lacticaseibacillus rhamnosus* with differentially enriched metabolites including lactic acid, ascorbic acid, and chlorogenic acid, which can desirably contribute to the flavor and quality of the red sour soup.

## 1. Introduction

Red sour soup is a traditional semisolid or liquid condiment product similar to ketchup but using different processing technology [1]. It is mainly famous in two of the major ethnic groups Miao and Dong living in the southwestern part of Guizhou Province in China. Furthermore, it has been a very popular traditional fermented soup in southwest China for thousands of years owing to its fascinating sour and spicy flavor [2]. It is generally made by fermenting nutritious raw materials including tomatoes, red chilli pepper, white wine, glutinous rice flour, bacterial species, and other ingredients [3]. It has a uniquely fresh, sweet, sour, and spicy flavor with a distinct aroma. It was designated as the Guizhou Graphical Indication Protection Product in 2013 due to its regional significance [4]. The product has extended outside Guizhou province in recent years and has become a popular hotpot component in a variety of Chinese cuisines. The red sour soup contains antioxidants, minerals, and vitamins and is a kind of functional food that helps to enhance the immune system, improve digestion, lower body fat content, and maintain healthy intestinal microbiota [5]. Traditional fermentation methods of producing red sour soup have some limitations which can be overcome by utilizing LAB inoculation and fermentation.

Fermentation with LAB produces lactic acid and other metabolites that play a variety of functions in the product’s overall quality. The fermentative ability of LAB makes them the most suitable fermenting agent owing to their desirable properties including nutrient enrichment, improvement in organoleptic characteristics, food safety, and health advantages [6]. It contributes considerably to the taste, texture, and in many cases, nutritive quality of food products [7]. LAB produces bacteriocins during fermentation, which are regarded as ‘natural inhibitors’ for other bacterial communities [7]. These bacteriocins serve as natural antimicrobial agents and increase the safety of food products. Apart from bacteriocins, LAB also produces metabolites that contribute to the flavor, taste, and odor of sour soup [8]. A recent study reported the presence of aldehydes and butyric acid as the causative agents of foul odor in sour soup [9]. Metagenomic (Illumina MiSeq) sequencing and metabolomic investigation of fermented Chinese sour soup detected 89 bacterial genera and 51 aromatic compounds (including 25 esters, eight alcohols, eight terpenes, three sulfur compounds, two acids, two ketones, one aldehyde, one pyrazine, and one monoterpene) in sour soup [8]. Metagenomic sequencing can be used to analyze millions of DNA sequences at the same time for the dissection of the genetic makeup of the microbial community to predict their functions [10].

The diversity of the core microbiota and the correlation between dominant bacteria and metabolites at different intervals of fermentation are important to understand the metabolic pathways and putative functional profile [11]. The metabolic activity of the bacterial community during fermentation changes with respect to time. The metabolites produced during this process contribute to food flavor, texture, and odor and serve as the major determinants of food quality [12]. Through metabolomic techniques, the metabolic contents of a biological sample could be thoroughly characterized, and metabolite profiles can be compared between samples [13]. Previous studies have determined the role of bacterial diversity in producing certain metabolites that were involved in producing a peculiar smell in the sour soup [8]. Microbial diversity of different Chinese fermented foods has been reported in different studies, however, the correlation between dominant bacterial species and primary metabolites in fermented red sour soup, is still unclear. Therefore, metagenomic and metabolomic studies of red sour soup are required to explore the association of bacterial species with the metabolite contents of the soup.

In the present study, the effect of LAB inoculation on the microbial diversity and metabolite contents at different fermentation times during the preparation of sour soup was investigated. We hypothesized that LAB inoculation can desirably affect bacterial diversity leading to the production of metabolites that can improve the nutrient composition, flavor, and aroma of the red soup.

## 2. Materials and Methods

### 2.1. Preparation of Sour Soup

The sour soup samples were prepared by pretreating 3.5 kg tomatoes and 0.5 kg chilli peppers in hot water. Stalks of tomatoes and peppers were removed followed by rinsing in water. After washing both vegetables, these were mixed in deionized water, minced, and grounded into a paste. About 1% salt, 1% wine, 0.75% ginger, 0.75% garlic, and 1% carbon source (glucose:lactose = 1:3 *w*/*w*) of the total volume of the mixture was added into the ground paste and stirred evenly. After mixing well, the mixture was put into a fermentation container.

### 2.2. Inoculant Fermentation

The dominant bacteria with a live bacterium count of 10^8^ CFU/mL (*C. intestinalis*:*L. rhamnosus*:*L. plantarum*:*L. casei*:*L. paracei* = 1:1:1:1:1) were added and fermented for 30 days in an incubator at 37 °C. Three replicates from each sample were randomly taken at 0 d, 5 d, 10 d, 15 d, 20 d, 25 d, and 30 d, respectively, with a total of 21 sour soup samples (Table 1). After collection, the samples were stored at −80 °C for further analysis. Details of samples fermented at different time intervals are given in Table 1.

### 2.3. DNA Extraction, PCR Amplification and Metagenomic Sequencing

The samples were taken at different time intervals to analyze their total DNA. The EZNA^®^ Soil DNA Kit (Omega Bio-tek, Inc., Norcross, GA, USA) was used to extract total DNA in the samples by following the manufacturer’s instructions. For the determination of DNA concentration, a Qubit^®^ dsDNA HS Assay Kit (Qubit v. 3.0, Hilden Germany) was used. For bacterial 16S rRNA amplification, the purified extracted DNA was utilized. The PCR primers used to amplify the V3 and V4 variable regions of 16S rDNA (“CCTACGGRRBGCASCAGKVRVGAAT” and “GGACTACNVGGGTWTCTAATCC”) were developed by GENEWIZ Company (GeneWiz, South Plainfield, NJ, USA). Additionally, PCR was used to add an index connector at the end of 16S rDNA for next-generation sequencing (NGS). PCR conditions for the amplification of DNA were: predenaturation for 3 min at 94 °C, denaturation for 5 s at 94 °C, annealing for 90 s at 57 °C, elongation for 10 s at 72 °C, and final elongation for 5 min at 72 °C. A total of 24 cycles of PCR amplification were completed by using 20 µL as a reaction mixture. Qubit v. 3.0 Fluorometer (Invitrogen, Carlsbad, CA, USA) was utilized to detect the library concentrations and quantified them to 10 nM. The PacBio sequencing platform (Illumina, San Diego, CA, USA) platform was used for the single-molecule real-time sequencing technology (SMRT) according to the manufacturer’s instructions.

The sequencing of replicated libraries of control and treatment samples fermented at different time intervals was performed by using PacBio Sequel/Sequel2 platforms, based on Single-Molecule Real-Time (SMRT) sequencing technology. To get a circular consistent sequence (CCS) or raw reads, the software SMRT Link v8.0 was used to analyze and filter the raw sequencing output data. Afterward, the raw reads were primed and length filtering (1300 to 1600 bp) was performed before the clean reads were used for further analysis (Table S1). For sequence clustering, VSEARCH (v. 1.9.6) was employed (the sequence similarity was set to 97%) using the Silva 132 as the 16S rRNA reference database. The OTU representative sequences were then subjected to a species assignment utilizing the RDP Classifier (Ribosomal Database Program) through the Bayesian method. Shannon, Chao1, and other diversity indices were derived based on the OTU analyses. For the normalized calculation of relative abundance of microbial communities, a *t*-test was performed using the SPSS 25.0 (IBM, Armonk, NY, USA). The Pearson’s correlation between bacteria and volatile flavor compounds was also calculated.

### 2.4. UPLC–MS/MS Conditions for Metabolome Analysis

For the metabolome analysis of sour soup samples, the Ultra Performance Liquid Chromatography (UPLC) (SHIMADZU Nexera X2, Kyoto, Japan), and Tandem mass spectrometry (MS/MS) (Applied Biosystems 4500 QTRAP, Waltham, MA, USA) was employed. After removing the soup samples from the −80 °C refrigerator, these were thawed, then vortexed for 10 s for mixing. An amount of 1 mL of the sample was taken in a 2 mL centrifuge tube, followed by the addition of 1000 μL of 70% methanol, and vortexed for 10 min. These samples were centrifuged for 10 min at 12,000 rpm/min (4 °C). The liquid was filtered with a microporous membrane (0.22 m) and stored in a sample bottle for LC–MS/MS testing.

#### 2.4.1. Liquid Phase Conditions

Agilent columns SB-C18 (1.8 sm, 2.1 mm × 100 mm) were used. The liquid phase consisted of the A phase for ultrapure water (added 0.1% of the acetic acid), and the B phase for acetylene (added 0.1% of the metformin). The gradient elution consisted of B phase ratio of 5% from 0 min to 9 min, B phase ratio with a linear increase to 95% and maintained from 10 min to 11 min, B phase ratio reduced to 5%, and balanced at 5% from 11 min to 14 min. The flow rate was 0.35 mL/min, the column temperature was 40 °C, and the sample volume of 4 μL were maintained.

#### 2.4.2. Conditions for Mass Spectrometry

LIT and triple quad pole (QQQ) scans were obtained using the Triple Quadlor Linear Ion Trap Mass Spectrometer (Q TRAP), ab4500 Q TRAP UPLC MS/MS system with the ESI Turbo Ion Spray Interface, which can be controlled by the Analyst 1.6.3 software (AB Sciex) to operate in both positive and negative ion modes. The following are the ESI source’s operating parameters: an ion source, turbine spray, source temperature 550 °C, ion spray voltage (IS) 5500 V (positive ion mode)/−4500 V (negative ion mode), ion source gas I (GSI), gas II (GSII), and curtain gas (CUR) were set to 50, 60, and 25.0 psi, respectively, and the collision induction electrode parameter was set to high. With 10 and 100 mol/L polypropylene glycol solutions, respectively, instrument tuning and mass calibration were done in QQQ and LIT modes. The collision gas (nitrogen) for QQQ scanning was set to medium and the MRM mode was used. The DP and CE of each MRM ion pair were completed by further optimization. Based on the metabolites eluted during each period, a specific set of MRM ion pairs was monitored at each period as reported previously [14].

### 2.5. Statistical Analysis

The statistics function prcomp in R software (www.r-project.org, accessed on 20 September 2021) was used to perform the unsupervised principal component analysis (PCA). Before the unsupervised PCA, autoscaling was done for each variable. The findings of Hierarchical Cluster Analysis (HCA) for samples and metabolites were shown as heatmaps with dendrograms using the R tool. The color spectrum of adjusted metabolite signal intensities (unit variance scaling) was displayed for HCA. The cor function in R was used to determine Pearson’s correlation coefficients between samples, and heatmaps were created.

VIP >= 1 and absolute Log2FC (fold change) >= 1 were used to identify differential metabolites between groups. The VIP values were obtained from the Orthogonal Least-multiplier Differential Analysis (OPLS-DA), which included score plots and permutation plots, and were constructed using the MetaboAnalystR software. Before OPLS-DA, the data were log-transformed (log2) and auto-scaled. A permutation test (200 permutations) was used to evaluate the OPLS-DA model, as reported earlier [15].

## 3. Results

### 3.1. Metagenomic Sequencing and Relative Abundance of Different Microbial Communities

The metagenomic libraries of control and LAB fermented samples were sequenced using PacBio SMRT sequencing technology and the raw sequence data were filtered and the alpha diversity indexes were calculated to examine the abundance and diversity of microbial species. The average length of sequences varied from 1431 to 1493 in different samples (Table S1). The OTU statistics based on the sequenced data revealed the species composition and relative abundance of each species. The relative abundance of the top 30 species revealed that three phyla (Firmicutes, Proteobacteria, and Bacteroidetes) constituted about 99.9% of the total bacterial population in all samples. In the control group (CK), Firmicutes was the major phyla (>80%) followed by the Proteobacteria (13%). After the LAB inoculation and the start of fermentation at T-I, about 99.9% abundance of phylum Firmicutes was observed. A slight increase in the abundance of Proteobacteria and Bacteroidetes was observed only at 15 days after fermentation (T-III) but afterward, the Firmicutes dominated over other phyla comprehensively from T-IV to T-VI (Figure S1).

**Figure 1:**
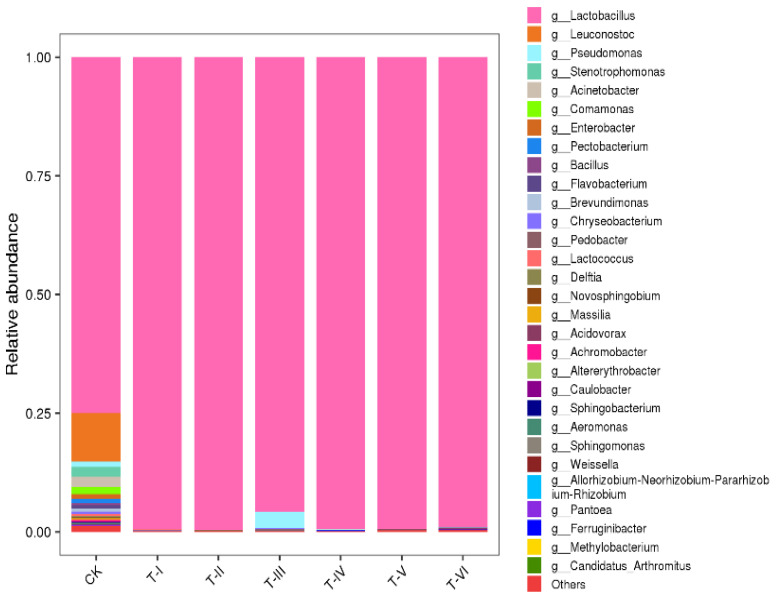
Relative abundance of bacterial genera in different soup samples (Sample codes indicate the samples fermented for 0 d (CK), 5 d (T-I), 10 d (T-II), 15 d (T-III), 20 d (T-IV), 25 d (T-V), and 30 d (T-VI)).

At the genus level, the results of samples without LAB inoculation (CK) revealed that the major 30 genera constituted about >99% of the total bacterial community, out of which 75% consisted of the *Lactobacillus* genus that dominated over the total microbial community. Right from the start of fermentation, *Lactobacillus* dominated over other bacterial species. There was a slight increase in the growth of *Pseudomonas* and *Leuconostoc* in the third interval (T-III) of the fermentation but, it again decreased after 20 days of fermentation (from T-IV to T-VI) as shown in Figure 1.

The heat map analysis of the top 12 most abundant phyla in the inoculated fermented samples revealed clear differences in the relative abundance of bacterial diversity between control and treatment groups. The predominant phyla showed variations in the relative abundance between control and treatment samples at different intervals. Particularly, phyla Deinococcus-Thermus, Proteobacteria, and Bacteroidetes showed clear variation and were higher in the CK group as compared to LAB fermented samples (T-I to T-VI). However, relative abundances of phyla Firmicutes and Planctomycetes were relatively highly abundant in treatment groups as compared to CK (Figure S2)

### 3.2. Alpha Diversity Indices of Bacterial Populations

The alpha diversity parameters including Chao1, ACE, Shannon, and Simpson were calculated in the present study. Results revealed significant effects of LAB inoculation on Shannon and Simpson indices as their value decreased (*p* < 0.01) with treatment at different time intervals (Table 2). However, Chao1 and ACE values did not show any significant difference among control and treatment samples.

### 3.3. Beta Diversity Analyses of Bacterial Composition (through NMDS, PCA and PCOA)

**Figure 2:**
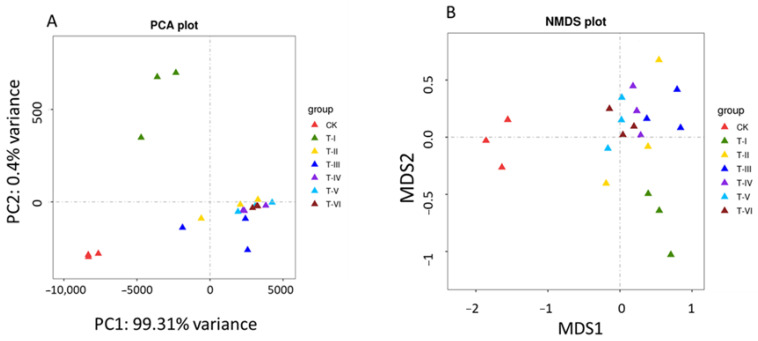
(**A**) PCA plot showing microbial diversity in groups, (**B**) Non-Metric Multidimensional Scaling (NMDS) plot showing variation in the microbial communities between different samples. (Sample codes indicate the samples fermented for 0 d (CK), 5 d (T-I), 10 d (T-II), 15 d (T-III), 20 d (T-IV), 25 d (T-V) and 30 d (T-VI)).

Principal Component Analysis (PCA) based on the relative abundance of microbial communities showed the difference in the bacterial community of LAB fermented samples (T-II, TIII, T-IV, T-V, and T-VI) that clustered closely to each other revealing distinct differences between CK and T samples. The first and second components of PCA (PC1 and PC2) explained 99.31% and 0.4% of the total variance, respectively (Figure 2A).

Non-Metric Multidimensional Scaling (NMDS) analysis based on Bray-Curtis dissimilarities was performed to compare the dissimilarities between bacterial composition in LAB fermented and control samples. Results revealed that samples from fermented sour soup (T-I to T-VI) were closely clustered in the NMDS plot while, control group (CK) samples were far apart from the LAB treated sample, revealing the clear differences in bacterial composition in the control and treatment samples (Figure 2B).

**Figure 3:**
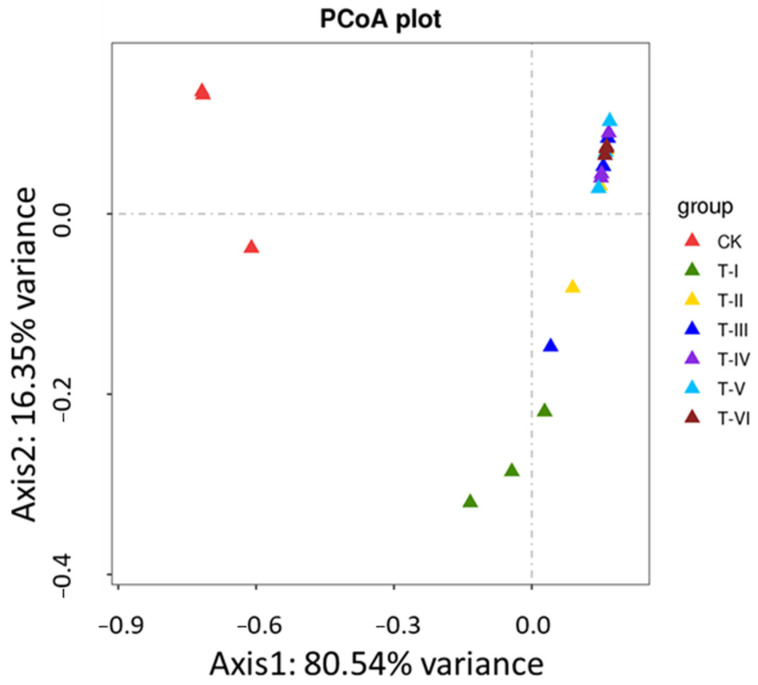
PCoA plot showing similarities and differences of potential main components at the evolutionary level in the samples. (Treatment codes indicate the samples fermented for 0 d (CK), 5 d (T-I), 10 d (T-II), 15 d (T-III), 20 d (T-IV), 25 d (T-V) and 30 d (T-VI).

Based on evolutionary distance, Unifrac PCoA plots are used to reveal the major components at the evolutionary level that affect the differences in community composition of different samples. The distance obtained in the Unifrac Analysis showed the similarity and differences in the samples. The PCoA plot, (Figure 3) exhibited that axis 1 and 2 explained 80.54% and 16.35% of the total variance in the samples, respectively, which indicates the difference in the composition of bacterial species in the control and different LAB fermented soup samples.

### 3.4. Network Map of Dominant Species and Venn Diagram Analysis

A network map was developed in which the node represents the species, the node of the red connection is positively correlated, the node of the green connection is negatively correlated, the size of the node is proportional to the abundance and proportionality of the species in all samples, and the node color represents the horizontal classification of the gate to which it belongs. Out of 12 phyla, only species from Firmicutes were negatively correlated and are shown in pink nodes in the network map (Figure S3).

**Figure 4:**
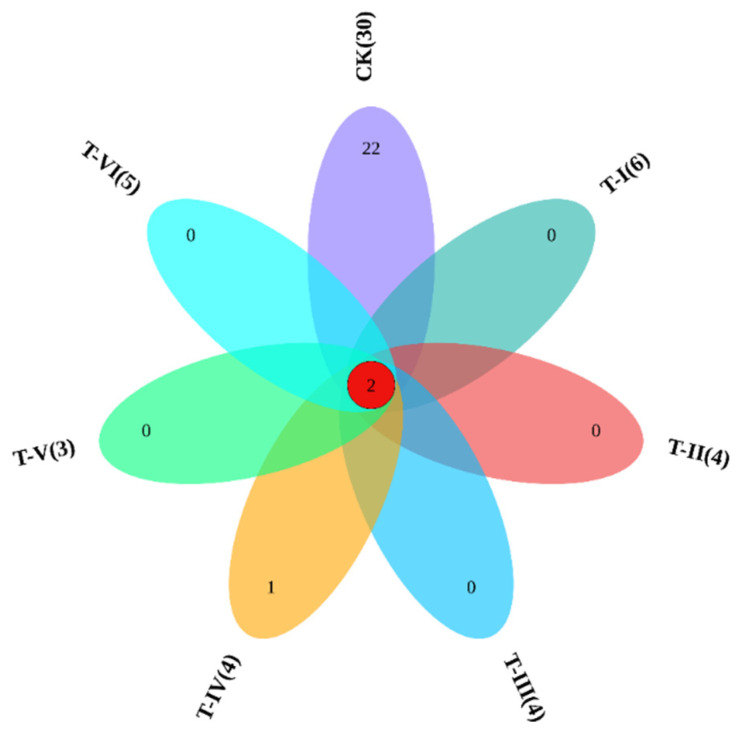
The Venn diagram showing common and unique OTUs in different samples.

The Venn diagram was plotted to reveal the common and unique OTUs among the control and different treatments groups of soup samples. Results revealed only two common bacterial species between the control and different treatment samples. However, there were 22 unique OTUs observed in the control while only one in the T-IV group (Figure 4).

### 3.5. LDA Effect Size (LEfSe) Analysis

To estimate the size of the effect of each component (species) abundance and their differences, LEfSe analysis was utilized through linear determination analysis (LDA) to identify predominant phyla in different groups. Results revealed a significant difference in LDA scores between different groups (Figure S4). The LDA scores indicated that bacterial species from phylum Firmicute were predominant in T-II, *Lacticaseibacillus* *rhamnosus* in the T-IV group and *Levilactobacillus* *brevis* in T-I.

### 3.6. Analysis of the Major Metabolic Products

**Figure 5:**
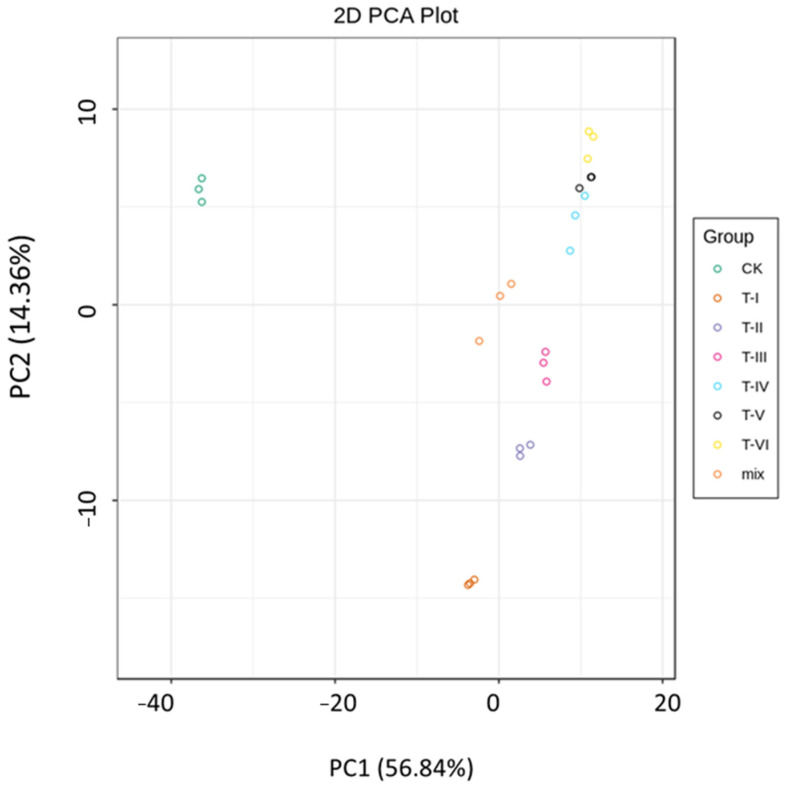
PCA plot showing the metabolomic difference between different groups.

The two-dimensional PCA revealed a clear separation of the control and treatment groups based on the presence of different metabolites. The first two principal components of PCA analysis PC1 (58.92%) and PC2 (14.97%) explained 73.89% of the total variance among samples (Figure 5). There was a clear separation of CK and T groups as they were clustered separately showing the presence of metabolic products at different fermentation intervals (Figure 5).

**Figure 6:**
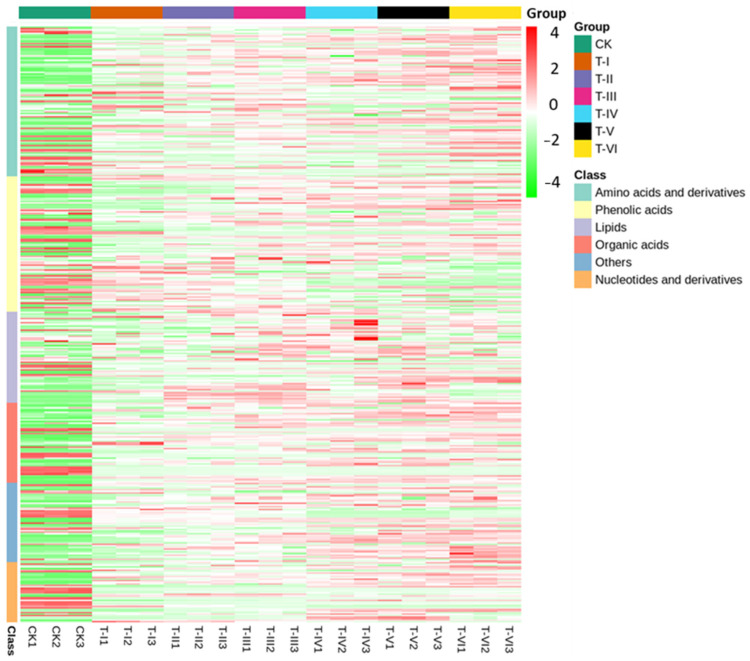
A heatmap graph showing clusters of metabolic compounds in different samples (Sample codes indicate the samples fermented for 0 d (CK), 5 d (T-I), 10 d (T-II), 15 d (T-III), 20 d (T-IV), 25 d (T-V), and 30 d (T-VI).

The Venn diagram showed 189 common in the CK and treatment groups in addition to one unique metabolite in T-III, two in T-IV, four in each T-V and T-VI (Figure S5). The heat maps of metabolites revealed the clusters of various metabolic compounds in different samples of CK and T groups. Overall results revealed the enrichment of different metabolites including amino acids and their derivatives after 20 days of fermentation. The intensity of lipid compounds in T-IV3 was very high, whereas amino acid and its derivatives compounds were more abundant in T-VI samples (Figure 6).

### 3.7. Orthogonal Least Multiplier-Differential Analysis (OPLS-DA)

The scores of each group were mapped using metabolite data and the OPLS-DA model, which further highlighted the variations between the groups. The T score and the orthogonal T score of CK with individual T groups of different samples revealed great variations among metabolites from respective groups. In comparison with CK, all T group samples showed an average orthogonal T score of 4.5% while the average T score was 82%. This difference showed clear variation between CK and T groups (T-I to T-VI) in terms of metabolite composition. Results of all comparisons between CK and T groups are presented in Figure S6A–F.

### 3.8. Screening of Differential Metabolites

The comparison of differential metabolites CK with all T groups (T-I to T-VI) (Figure S7), revealed a significant increase in the relative content of 10 metabolites namely Lmrn002746 (2-hydroxy-4-methylpentanoic acid), pme2237 (dulcitol), Lmrn003201 (indole-3-lactic acid), mws0341 (2-hydroxyisocaproic acid), mws0251 (thymine), Hmfn000531 (l-ascorbic acid Vitamin C), pme3382 (*N*-acetyl-l-threonine), mws0467 (3-4-hydroxyphenyl-propionic acid), pmb0530 (nicotinic acid adenine dinucleotide), lmrj001341(cyclo-ser-pro). There was a decrease in the relative content of 10 metabolites in comparison to CK with all T groups. Out of these 10 metabolites, mws0159 (l-homocitrulline) was significantly reduced in the treatment groups.

### 3.9. KEGG Classification and Differential Metabolite Enrichment Analysis

**Figure 7:**
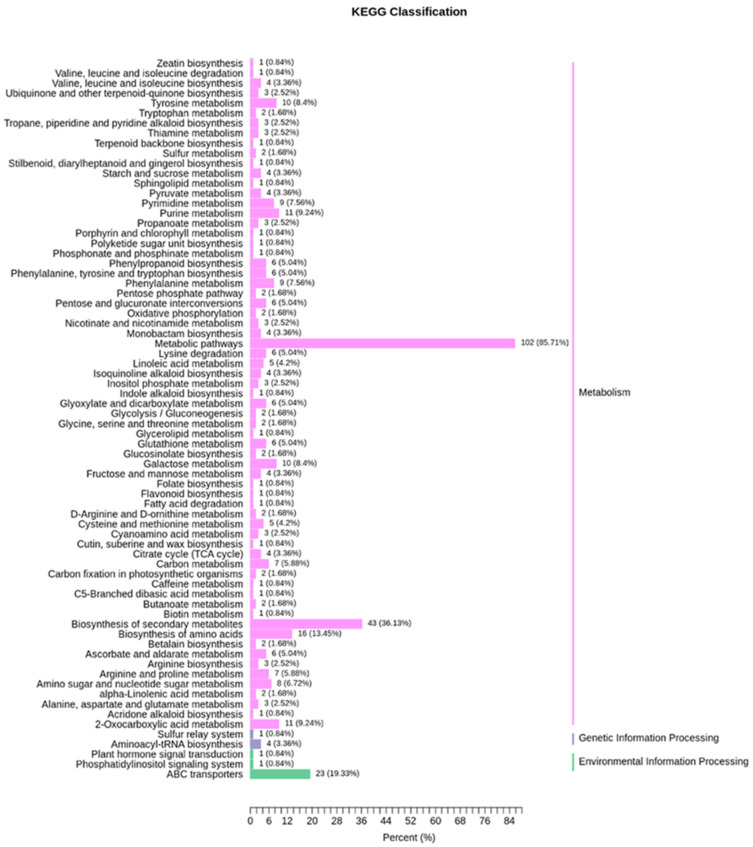
Comparison of KEGG metabolic pathway classification between CK and T groups.

The KEGG enrichment analysis was carried out on the differential metabolites to better understand the underlying metabolic pathways. Out of 393 metabolites identified in all the samples, 69 metabolic pathways were classified and annotated through KEGG. Compared to the control group, 10 significantly enriched KEGG metabolic pathways were observed in treatment groups including, metabolic pathways, biosynthesis of secondary metabolites, biosynthesis of amino acids, amino sugar and nucleotide sugar metabolism, 2-oxocarboxylic acid metabolism, ABC transporters, galactose metabolism, purine metabolism, pyrimidine metabolism, and tyrosine metabolism (Figure 7).

KEGG enrichment statistics showed the comparison of relative contents of differential metabolites at different stages of fermentation. The 20 most enriched differential metabolites were presented with their *p*-values and richness factor (Figure S8).

**Figure 8:**
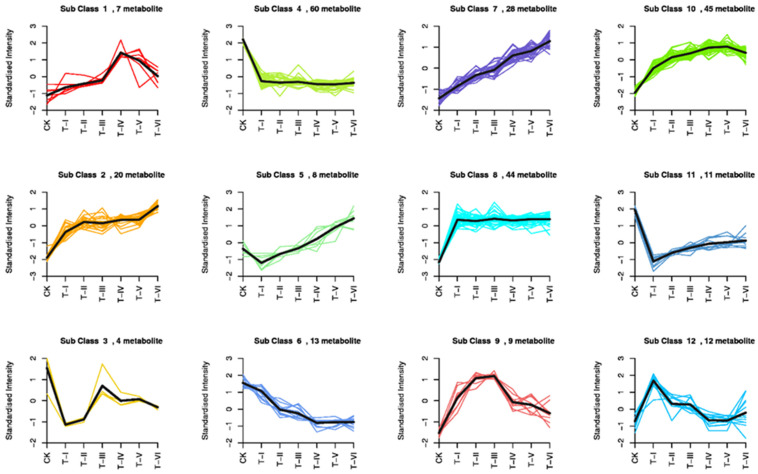
Results of k-means clustering analysis for different treatment groups. Treatment codes indicate the samples fermented for 0 d (CK), 5 d (T-I), 10 d (T-II), 15 d (T-III), 20 d (T-IV), 25 d (T-V), and 30 d (T-VI).

The relative content of differential metabolites (z-score) was standardized for each group, and then the k-means clustering was conducted to evaluate the trend of relative content variations of the metabolites in distinct groups. Variation of different sub-classes of metabolites in different groups was studied using k-means analysis. There were 60 metabolites in sub-class 4 and their standard intensity was decreased after the start of fermentation whereas, in sub-class 7, 28 metabolites showed an increasing trend during the fermentation period (Figure 8).

### 3.10. Correlation between Dominant Bacteria and Primary Metabolites

**Figure 9:**
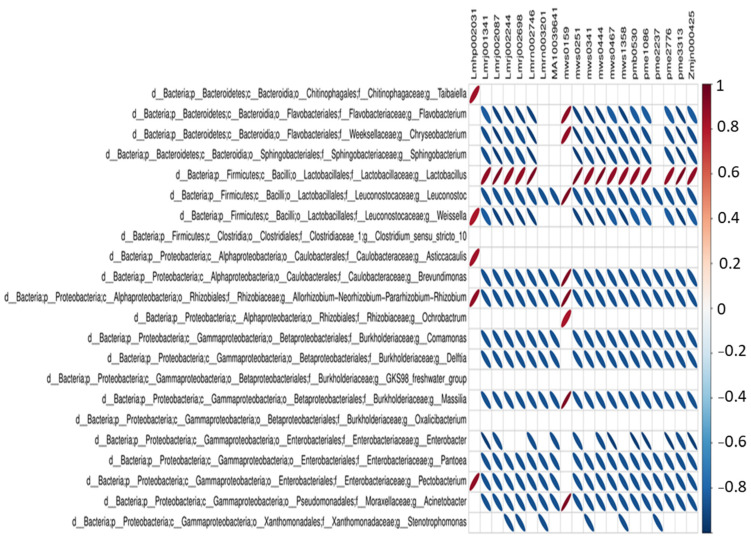
Heat map showing the Spearman correlation of the top 20 differential metabolites and dominant bacterial genera (A red ellipse indicates positive correlation, while blue ellipse represents a negative correlation. The higher the absolute value of the correlation, the thinner the ellipse. A blank grid indicates that the significance *p* value is greater than 0.05).

Results of the Spearman correlation analysis among dominant bacteria and the top 20 primary metabolites revealed a very strong positive correlation of *Lactobacillus* bacteria with 15 primary metabolites out of 20 in fermented samples (Figure 9). *Lactobacillus* was strongly positively correlated with amino acid (l-isoleucyl-l-aspartate), organic acid (2-hydroxy-4-methylpentanoic acid), nucleotide derivatives (thymine), phenolic acid (3-aminosalicylic acid), sugars, and alcohols (d-fructose 6-phosphate). The correlation network analysis of dominant bacteria is presented in Figure S9.

**Figure 10:**
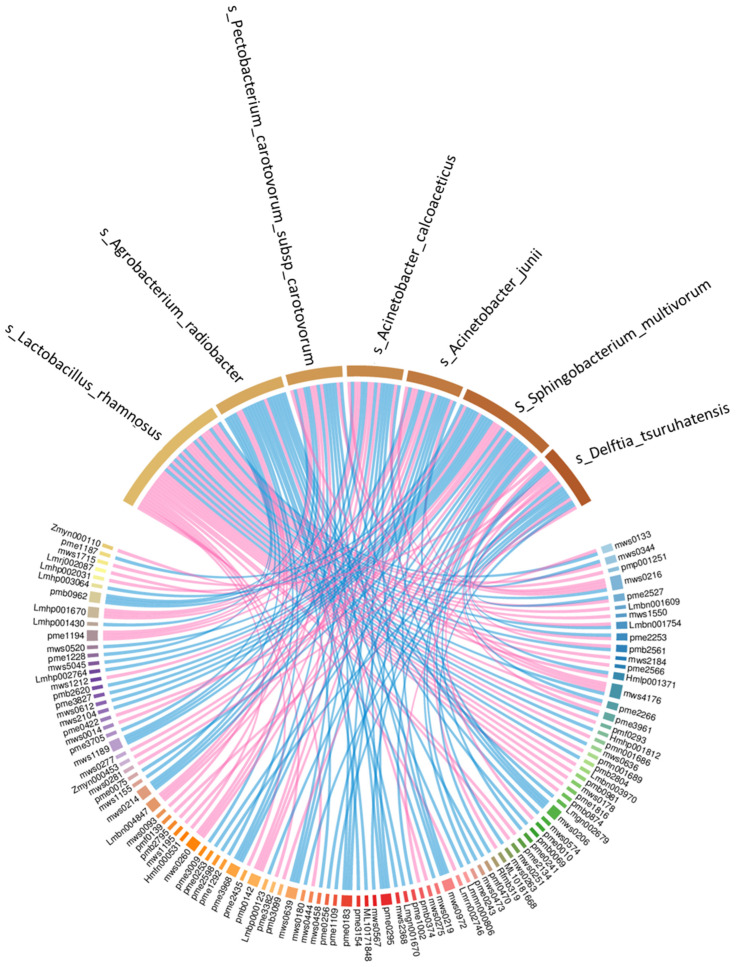
Chord network diagram of the correlation between dominant bacterial species and significant metabolites (The width of the string connection represents the size of the correlation between the two variables connected, and the wider the link, the greater the absolute value of the correlation while, pink string indicates a positive correlation and blue indicates a negative correlation).

The correlation network diagram of dominant bacterial species with top enriched metabolites is shown in Figure 10. The Lacticaseibacillus palm species positively correlated with d-xylonic acid (Mws0344), lysoPC 18:2(2n isomer) (pmp001251), l-ornithine (pme2527), 2-acetyl-2-hydroxybutanoic Acid (lmbn001609), S-allyl-l-cysteine (mws1550), l-gulono-1,4-lactone (pme2253), ethyl caffeate (mws2184), 5-l-glutamyl-l-amino acid (pme2566), cyclo(Tyr-Ala) (hmlp001371), biotin (pme2266), 2′-deoxyadenosine (pme3961), 1-octadecanol (pmf0293), 10,16-dihydroxypalmitic acid (pmn001686), 9-hydroxy-12-oxo-15(Z)-octadecenoic acid (pmn001689), 9,12,13-TriHOME; 9(S),12(S),13(S)-trihydroxy-10(E)-octadecenoic acid (lmbn003970), adenosine 5′-monophosphate (pmb0981), chlorogenic acid (3-*O*-Caffeoylquinic acid) (mws0178), neochlorogenic acid (5-*O*-caffeoylquinic acid) (pme1816), lysoPE 18:2(2n isomer) (pmb0874), d-threitol (pme2134), thymine (mws0251), 2-hydroxy-4-methylpentanoic acid (lmrn002746), 3-aminosalicylic acid (mws0444), *N*-acetyl-l-threonine (pme3382), l-fucose (pme2435) and l-ascorbic acid (Vitamin C) (hmfn000531). Lacticaseibacillus rhamnosus is negatively correlated with nicotinamide (mws0133), trans-4-hydroxy-l-proline (mws0216), 3-isopropylmalic acid (lmbn001754), *N*-acetyl-l-methionine (pmb2561), l-alanyl-l-phenylalanine (mws4176), 2′-deoxyadenosine (pme3961), 2′-*O*-methyladenosine (hmhp001812), l-phenylalanyl-l-phenylalanine (mws0636) and 13S-hydroperoxy-9Z,11E-octadecadienoic acid (pmb2804).

## 4. Discussion

The present study aimed to evaluate the potential of LAB inoculation as a starter culture for sour soup fermentation. Previously, *Lactobacillus* species have been used in the fermentation of different vegetables and fruits [16]. *Lactobacillus* species play an important role for the desirable fermentation of foods without affecting the quality of fermented products owing to their anaerobic metabolism. Their metabolites not only enrich the fermented foods with nutrients but also hinder the growth of undesirable bacteria that spoil the food or deteriorate the quality of fermented products [17,18,19].

In the present study, the bacterial diversity and metabolomic analyses revealed variation in the number and diversity of bacteria in LAB fermented sour soup at different intervals of fermentation. The relative abundance of different bacterial taxa in the control and fermented samples revealed that LAB consistently remained dominant throughout the fermentation period and their number increased after the inoculation.

Major abundant species during fermentation were mostly from phyla Firmicutes, Proteobacteria, and Bacteroidetes. By using heatmap clustering, PCA, PCoA, NMDS, and Venn diagram analysis, we observed clear separation of control and fermented samples at different intervals of fermentation. This indicates the advantageous role of LAB as a starter culture to desirably affect the microbial contents of fermented foods [20,21]. Similar findings have been reported earlier by using *L. bucheneri* H9 as an inoculant for the rapid fermentation of red sour soup [5]. As we used DNA samples in the present study, which could not discriminate between metabolically active or inactive microorganisms that is somehow a limitation of the present study. A culture-dependent approach and/or pyrosequencing based on RNA samples would have helped in defining the actual bacterial community in the fermented samples, moreover, a control group without LAB inoculation might have facilitated the native microbes to grow in the fermented soup which would be considered in future studies to elucidate influence of LAB on fermentation process.

The correlation network demonstrated the negative correlation of LAB with other bacterial species revealing that an increase in the number of LAB leads to a subsequent decrease in other bacterial strains in the fermented soup. Bacteriocins produced by LAB with broad-spectrum antibacterial action can enhance food safety, microflora, product shelf life, and suppress pathogenic bacterial growth, all of which improve the quality of fermented food [22,23,24]. The LAB fermentation can produce lactic acid much faster leading to a significant shortening of the fermentation period of red sour soup preparation. Furthermore, faster lactic acid production also facilitates the growth inhibition of miscellaneous bacteria during the early stage of fermentation [5,25], which was quite evident from LAB counts observed in the control and treatment samples. Moreover, the negative correlation of LAB with other bacterial strains observed in this study is also consistent with these findings. In the present study, the LAB count reached 99% after just five days of fermentation but it was only 75% in the control group, revealing the speed of lactic acid production and wiping out of other bacterial stains ensuring desirable flavor and metabolites in the fermented soup. Similar findings were observed in the preparation of indigenous food mixtures containing tomato pulp through inoculation with *L. casei* and *L. plantarum*. The fermentation with these LAB strains resulted in a sharp decrease in pH, an increase in acidity, and an improvement in the digestibility of starch and protein contents [26].

In the current study, the effect of LAB inoculation on the production of metabolites was also investigated through a metabolomic analysis. Results of PCA and heatmap analyses revealed the significant variation in metabolite composition in the LAB fermented samples. The KEGG classification of differential metabolites revealed that enriched metabolites were mainly amino acids and their derivatives, organic compounds, lipid compounds, and nucleotides and their derivatives. Out of 393 metabolites, 144 different metabolites were identified and mainly included amino acids and their derivatives, nucleotides and their derivatives, alkaloids, sugars and alcohols, phenolic acids, organic acids, and other secondary metabolites. Previous studies have also shown that taste substances in the inoculated group were more abundant and the intensity of the sourness was highest owing to the dynamics from saccharification to alcoholization and acidification in the fermentation process of foods inoculated with LAB and yeasts [27,28]. A recent study reported the correlation between microbial communities (bacterial and fungal) and important volatile metabolites in red pepper (*Capsicum annuum* L.) fermentation [29].

The present study indicated that *Lactobacillus* was the most predominant and significant bacterial taxa during the fermentation of red sour soup. The positive association of *Lactobacillus* with some of the important metabolites like chlorogenic acid (3-*O*-caffeoylquinic acid) was observed. Chlorogenic acid is a phenylpropanoid compound and plays many key physiological roles such as antioxidant, anticancer, antibacterial, antihistaminic, and other health promoting effects [30]. *Lactobacillus* inoculation also increases the production of l-ascorbic acid (Vitamin-C), which has many health benefits in humans like strengthening the immune system, the primary ingredient of collagen [31], assisting the biosynthesis of the carnitine and catecholamine amino acids that regulate the nervous system [32] and also facilitate the metabolism of fats and protecting tissues from free radical damages to support cardiovascular system [33]. Aspartic acid is a non-essential amino acid that helps in hormone synthesis and facilitates the normal nervous system (Sánchez and Vázquez, 2017). *Lactobacillu*s also stimulates the production of l-ornithine which helps in stress relief and increases sleep quality [34]. These facts indicate that LAB fermentation can enhance nutritive and health promoting value of fermented foods.

In the present study, the KEGG enrichment analysis revealed that three top enriched metabolic pathways included biosynthesis of secondary metabolites, biosynthesis of amino acids, amino sugar and nucleotide sugar metabolism in LAB inoculated soup samples. The amino acid metabolism is very crucial in the formation of metabolites that impart taste to fermented foods. This is mainly attributed to the formation of α-keto acids from the produced amino acids by aminotransferases. The synthesis of α-keto acids is the first step in the production of taste metabolites [35]. For example, leucine is involved in the production of 3-methylbutanoic acid, and deamination of serine might supply pyruvate, which can be subsequently converted into acetic acid, 2,3-butanedione, and acetoin [36]. These findings indicated that amino acid metabolism is not only the starting point for the synthesis of various metabolites such as glycine, cysteine, and serine phospholipids but also supplies essential amino acids for protein synthesis [37]. Moreover, most amino acids are essential metabolites that are required for the growth and protein synthesis of LAB in the fermentation process [28]. Our findings also indicated that mixed inoculation of different LAB strains exhibited synergistic effects on the fermentation process and production of metabolites, which is in agreement with earlier studies [28]. Collectively, these findings indicate the effectiveness of enriched pathways (particularly amino acid metabolism) in LAB fermented soup to have a more pleasant taste, flavor, and nutrient profile. Therefore, LAB inoculation can be sought as a desirable strategy to speed up the process of red sour soup formation while maintaining the optimum quality and nutrition.

## 5. Conclusions

The bacterial diversity in normal sour soup was higher than in LAB fermented soup samples. The total number of mesophilic microorganisms and LAB community was dominating which indicated that LAB inoculation imparted a significant effect on the microbial diversity and metabolic contents produced during the fermentation of red sour soup. The increased content of 10 metabolites in fermented soup samples (particularly lactic acid, thymine, and ascorbic acid) were observed. The correlation network revealed positive association of *Lacticaseibacillus rhamnosus* with differentially enriched metabolites including lactic acid, ascorbic acid, and chlorogenic acid, which can desirably affect the flavor and quality of the red sour soup. Further investigations are warranted to explore the molecular mechanisms of formation and metabolic pathways of metabolites affecting flavors in the fermented soup under the conditions of different fermentation times.

## Figures and Tables

**Table 1:** Details of red sour soup samples fermented for different intervals.

Treatment Code	Fermentation Period
CK	Samples fermented for 0 Days
T-I	Samples fermented for 5 Days
T-II	Samples fermented for 10 Days
T-III	Samples fermented for 15 Days
T-IV	Samples fermented for 20 Days
T-V	Samples fermented for 25 Days
T-VI	Samples fermented for 30 Days

**Table 2:** Alpha diversity indices of microbial communities.

Parameter	CK	T-I	T-II	T-III	T-IV	T-V	T-VI	SEM	*p* Value
Chao1	80.37	49.14	80.71	46.72	63.22	74.50	58.93	15.10	0.53
ACE	82.21	50.14	77.40	47.84	66.43	75.07	68.39	11.81	0.33
Observed Species	49.00 ^a^	24.33 ^b^	31.33 ^ab^	26.33 ^b^	34.33 ^ab^	43.33 ^ab^	47.00 ^a^	4.21	0.001
Shannon	1.79 ^a^	0.70 ^c^	0.10 ^c^	0.29 ^bc^	0.10 ^c^	0.11 ^c^	0.15 ^c^	0.10 ^c^	0.001
Simpson	0.43 ^a^	0.24 ^b^	0.02 ^c^	0.08 ^c^	0.02 ^c^	0.02 ^c^	0.02 ^c^	0.03	0.001

Note: The values in the same row with different superscripts differ significantly (*p* < 0.05).

## Data Availability

Research data are not shared.

## Supplementary Materials

The following are available online at https://www.mdpi.com/article/10.3390/foods11030341/s1, Figure S1: Relative abundance of bacterial phyla in different soup samples, Figure S2: A heatmap showing clusters of different phyla in different groups, Figure S3: Associated network map showing positive and negative correlations between bacterial species, Figure S4: LDA Effect Size (LEfSe) analysis showing pre-dominant bacterial strain in each treatment group, Figure S5: Venn diagram showing common and unique metabolites in the control and different treatment samples, Figure S6: OPLS-DA plots showing variations in metabolites in different treatment groups, Figure S7: Differential metabolite screening bar charts, Figure S8: KEGG enrichment statistics and Figure S9: Heat map showing Spearman correlation of top 20 differential metabolites and dominant bacterial species, Table S1: Sequencing data statistics.
