# Supplementary material for: The Correlation Mechanism between Dominant Bacteria and Primary Metabolites during Fermentation of Red Sour Soup
Source: Foods. 2022 Jan 25;11(3):341. doi: 10.3390/foods11030341 (PMC8833966; doi:10.3390/foods11030341)
Supplement: Supplementary file 1 [file foods-11-00341-s001.zip › foods-1539498-supplementary.pdf]

Table S1. Sequencing data statistics.

Sample	CCS	Non-Primers	Filtered	Average Length
**CK1**	14509	13896	12257	1431
**CK2**	7735	7308	6424	1431
**CK3**	17292	13276	11729	1427
**T-I1**	7099	6631	6624	1491
**T-I2**	5418	5100	5097	1492
**T-I3**	8460	7951	7945	1491
**T-II1**	9143	8569	8565	1492
**T-II2**	13253	12527	12523	1492
**T-II3**	12119	11396	11389	1492
**T-III1**	13550	13031	13025	1489
**T-III2**	7805	7255	7248	1493
**T-III3**	12302	11829	11829	1492
**T-IV1**	11854	11397	11394	1493
**T-IV2**	12048	11561	11554	1492
**T-IV3**	13545	13019	13018	1492
**T-V1**	13963	13523	13518	1492
**T-V2**	11520	11080	11074	1493
**T-V3**	13021	12284	12278	1492
**T-VI1**	12742	12178	12171	1492
**T-VI2**	13078	12531	12527	1492
**T-VI3**	13037	12453	12450	1492

Table description: (1st row) Samples name, (2nd row) Number of original CCS sequences, (3rd row) Number of CCS after primer removal, (4th row) Number of valid CCS sequences after length filtering, (5th row) Average length of valid sequences.

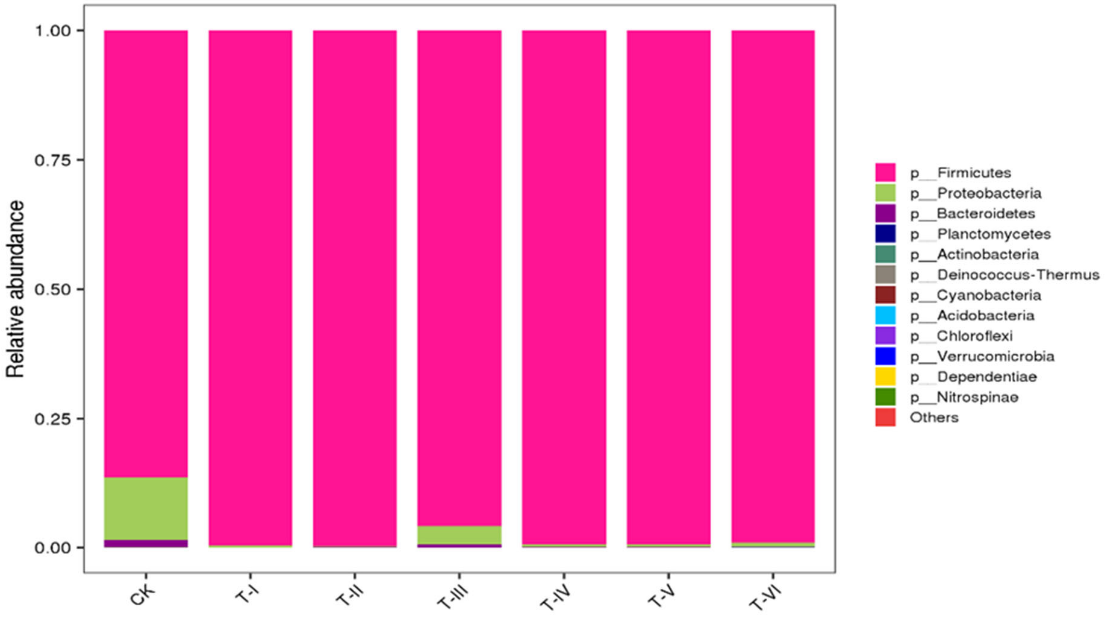

Figure S1. Relative abundance of bacterial phyla in different soup samples (Samples codes indicate the samples fermented for 0d (CK), 5d (T-I), 10d (T-II), 15d (T-III), 20d (T-IV), 25d (T-V), and 30d (T-VI).

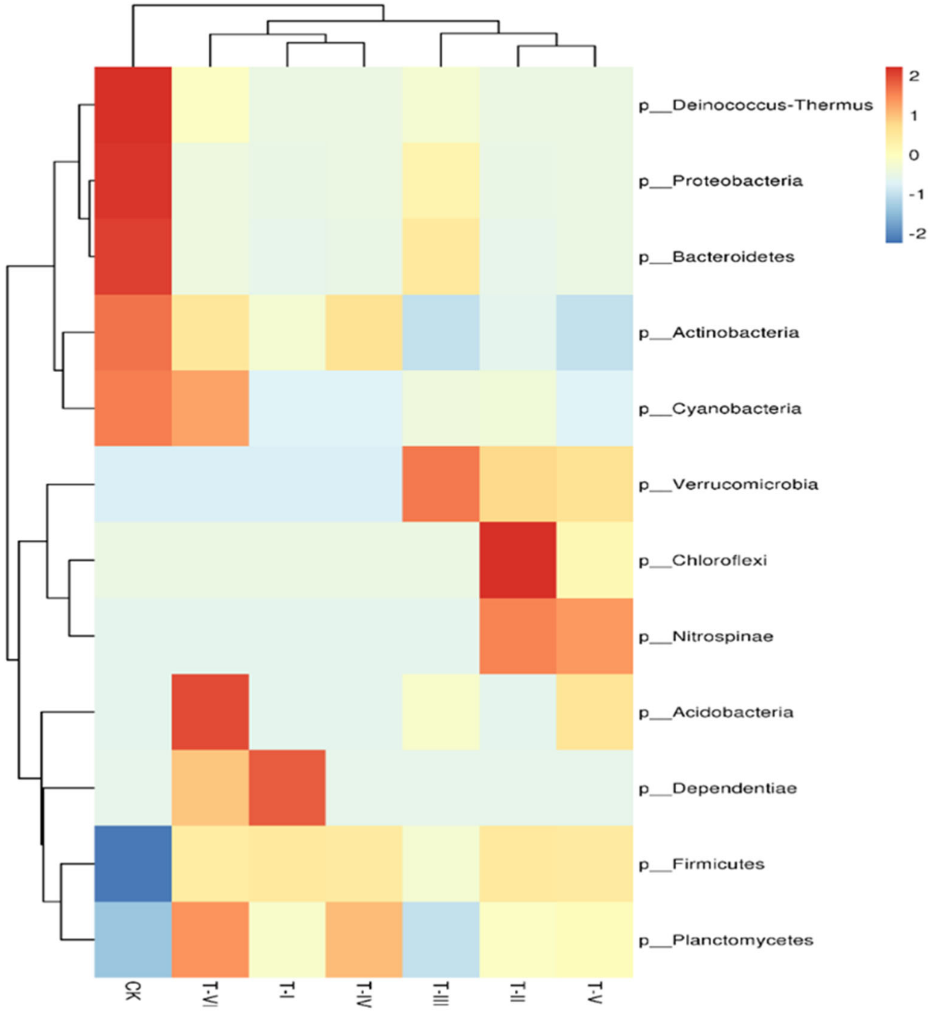

Figure S2. A heatmap showing clusters of different phyla in different groups. (Samples' codes indicate the samples fermented for 0d (CK), 5d (T-I), 10d (T-II), 15d (T-III), 20d (T-IV), 25d (T-V) and 30d (T-VI).

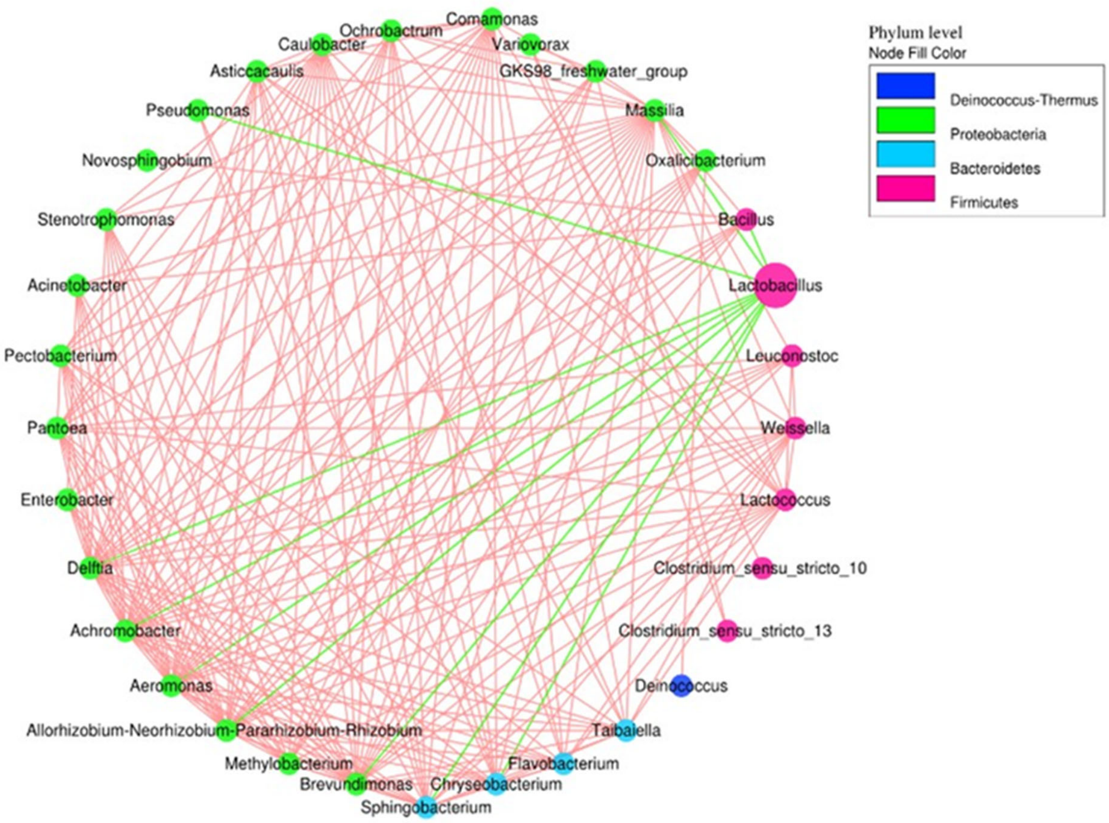

Figure S3. Associated network map showing positive and negative correlations between bacterial species.

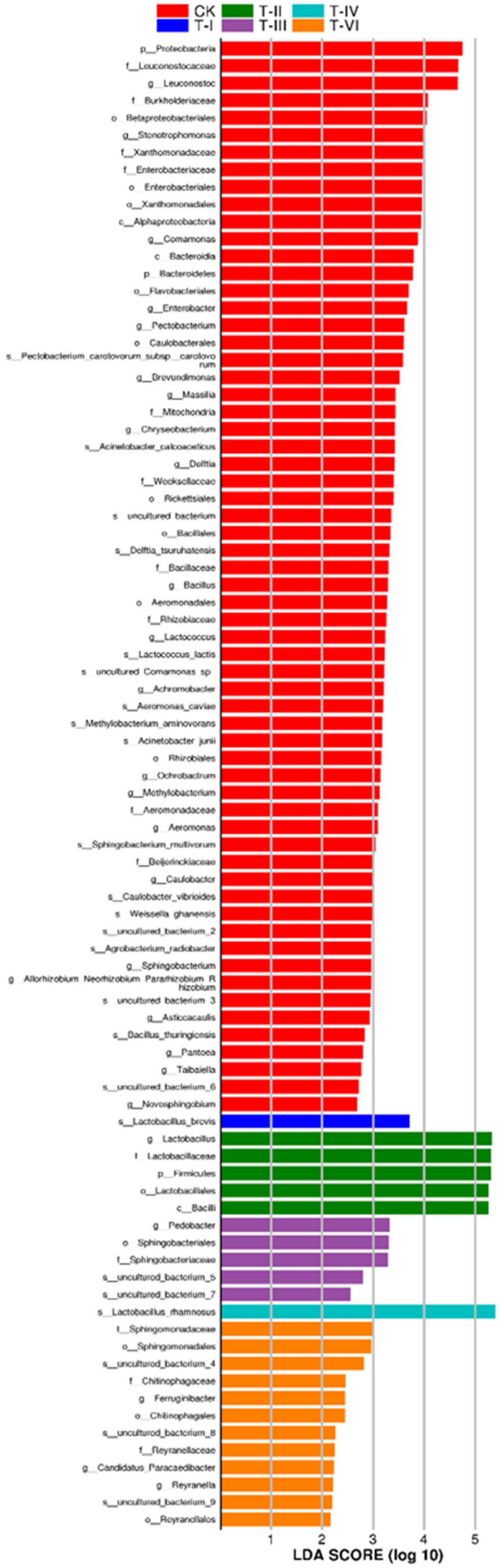

Figure S4. LDA Effect Size (LEfSe) analysis showing pre-dominant bacterial strain in each treatment group.

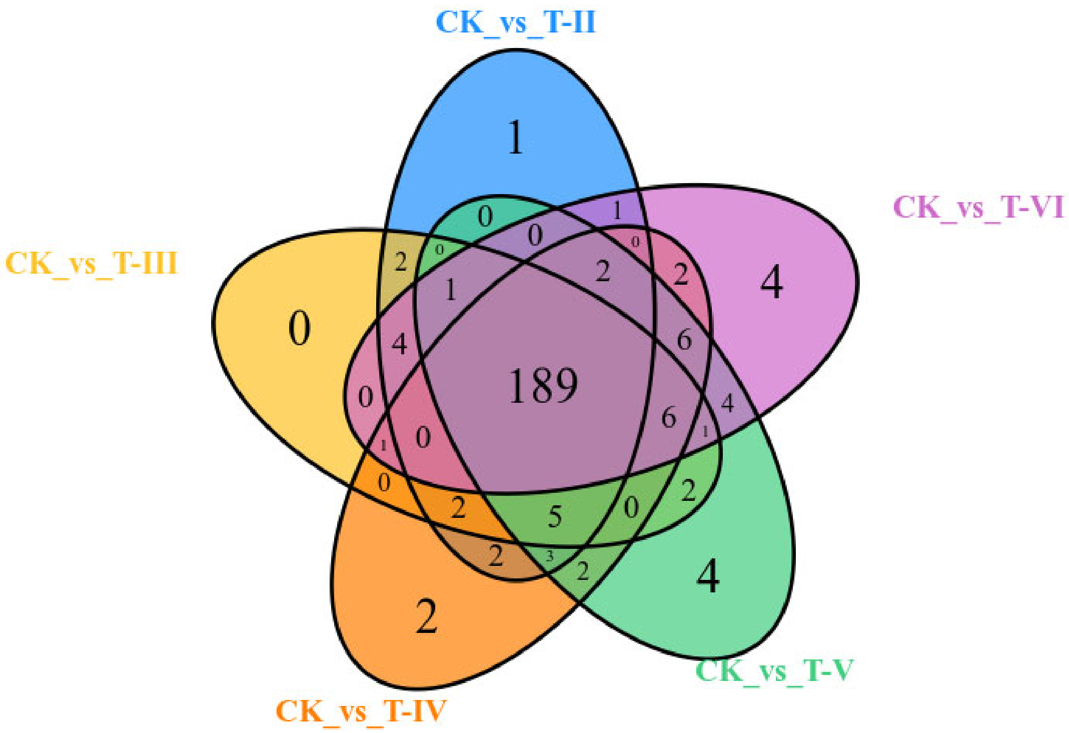

Figure S5. Venn diagram showing common and unique metabolites in the control and different treatment samples.

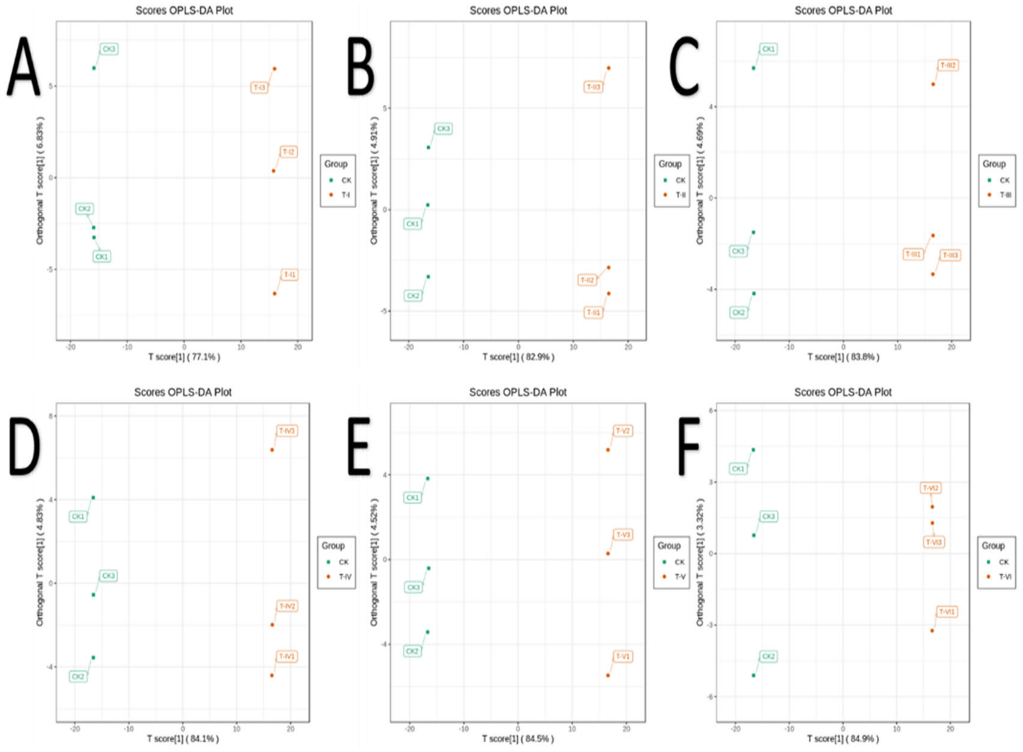

Figure S6. A-F. OPLS-DA plots showing variations in metabolites in different treatment groups. (Sample's codes indicate the samples fermented for 0d (CK), 5d (T-I), 10d (T-II), 15d (T-III), 20d (T-IV), 25d (T-V) and 30d (T-VI).

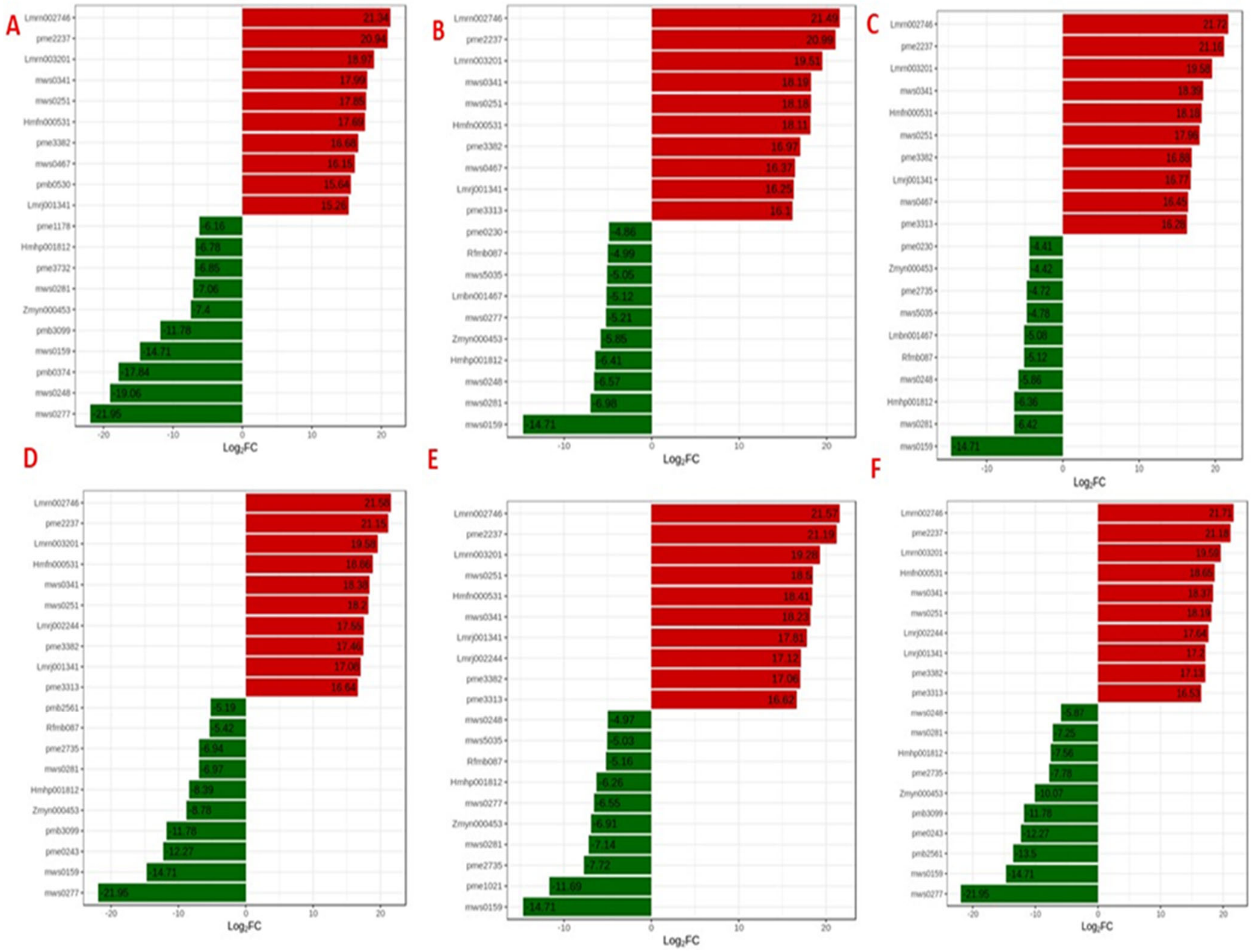

Figure S7. (A) Differential metabolite screening bar chart, CK vs T-I, (B) Differential metabolite screening bar chart, CK vs T-II; (C) Differential metabolite screening bar chart, CK vs T-III; (D) Differential metabolite screening bar chart, CK vs T-IV; (E) Differential metabolite screening bar chart, CK vs T-V; (F) Differential metabolite screening bar chart, CK vs T-VI**.** The p-value indicates the richness of differential metabolites in the group, dark red points showed the extent of significant enrichment of metabolites in that particular group, and the size of the point indicated the number of different metabolites enriched.

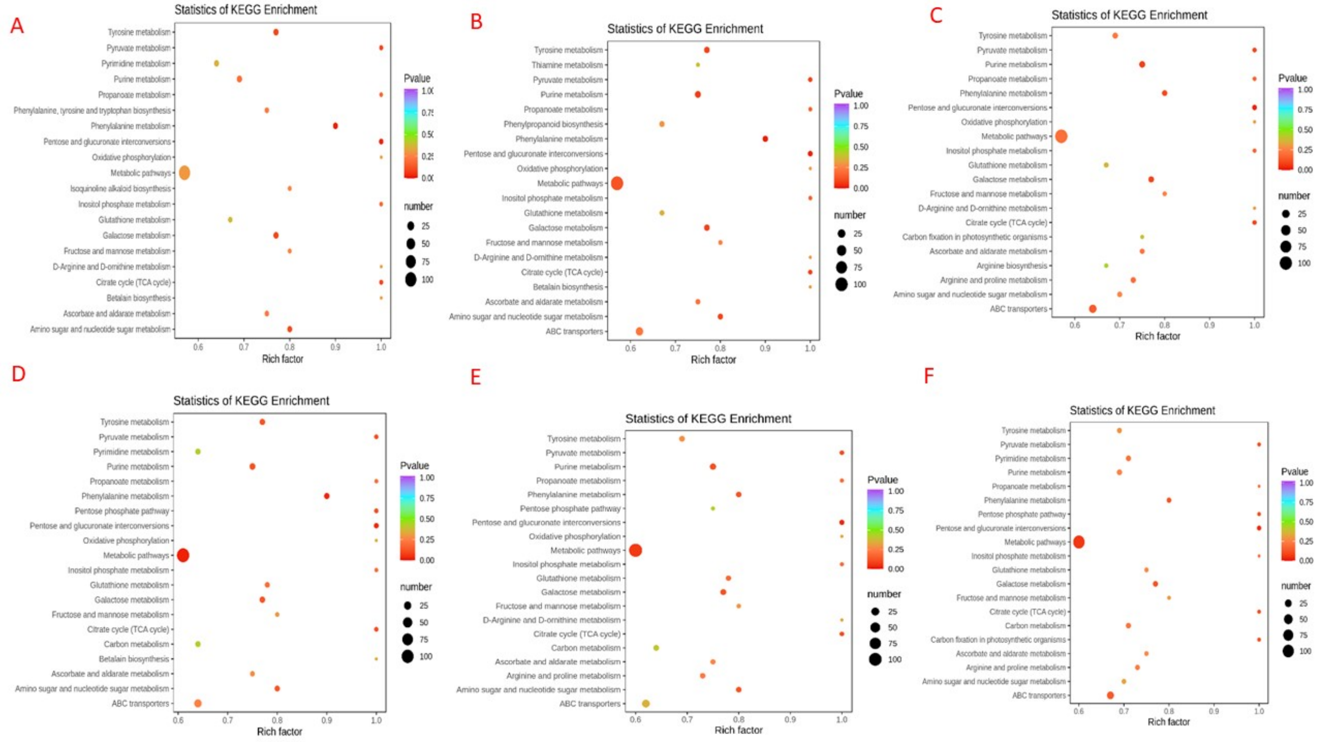

Figure S8. A:KEGG enrichment statistics in CK vs T-I; B:CK vs T-II; C CK vs T-III; D: CK vs T-IV; E:CK vs T-V; CK vs T-VI.

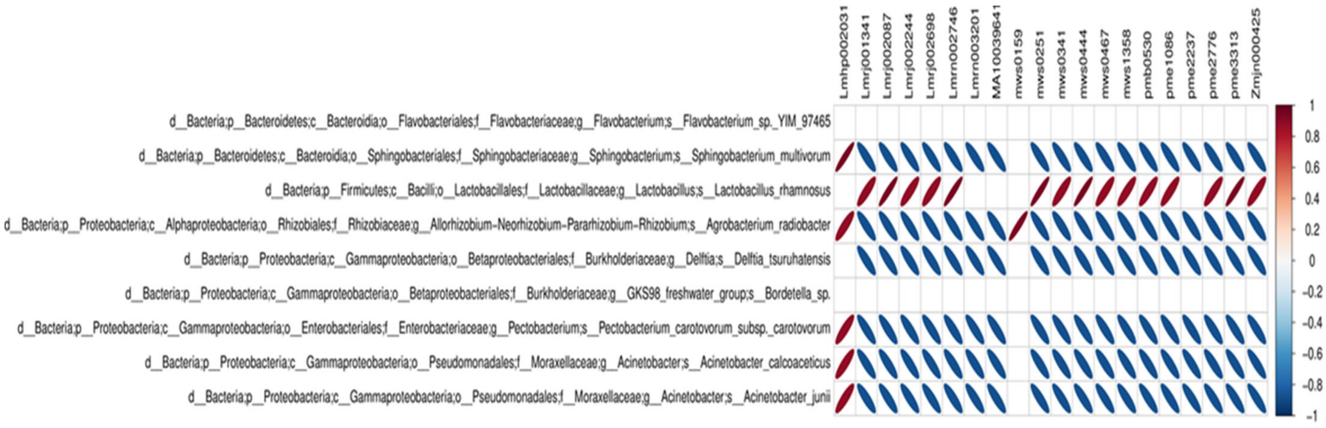

Figure S9. Heat map showing Spearman correlation of top 20 differential metabolites and dominant bacterial species (A red ellipse indicates positive correlation, while blue ellipse represents a negative correlation. The higher the absolute value of the correlation, the thinner the ellipse. A blank grid indicates that the significance P value is greater than 0.05).
